# Treatment of vascular risk factors in patients with a diagnosis of Alzheimer’s disease: a systematic review

**DOI:** 10.1186/s12916-014-0160-z

**Published:** 2014-11-11

**Authors:** Raffaella Valenti, Leonardo Pantoni, Hugh S Markus

**Affiliations:** NEUROFARBA Department, Neuroscience Section, University of Florence, Florence, Italy; Stroke Unit and Neurology, Azienda Ospedaliero Universitaria Careggi, Florence, Italy; Department of Clinical Neurosciences, University of Cambridge, Cambridge, UK

**Keywords:** Alzheimer’s disease, Cholesterol, Dementia, Diabetes mellitus, Hypertension, Progression, Smoking, Statin, Treatment, Vascular risk factors

## Abstract

**Background:**

Increasing evidence suggests vascular risk factors (VRF) play a role in the pathogenesis of Alzheimer’s disease (AD). Epidemiological studies have found associations between VRF and risk of AD. Treating VRF in patients with AD offers a potential treatment option but ineffective treatments should be avoided in this group who are frequently on multiple medications and in whom compliance may be challenging.

**Methods:**

Studies containing information on the treatment of VRF in patients with a diagnosis of AD were identified using a defined search strategy. Randomised controlled trials and observational studies were included.

**Results:**

The pre-specified search strategy retrieved 11,992 abstract articles, and 25 papers including those identified on review of reference lists and reviews met the inclusion criteria. Of these, 11 were randomised controlled trials (RCTs) and 14 observational studies. Observational studies suggested that a VRF package and treatment of hypertension and statin therapy may be associated with improved outcome but these studies suffered from potential bias. The few RCTs performed were mostly small with short duration follow-up, and do not provide clear evidence either way.

**Conclusions:**

Observational data raises the possibility that treating VRF could alter the rate of decline in AD. However RCT data are not yet available to support this hypothesis and to alter clinical practice. RCTs in larger numbers of individuals with longer follow-up, ideally in the early stages of AD, are required to address this potentially important treatment question.

**Electronic supplementary material:**

The online version of this article (doi:10.1186/s12916-014-0160-z) contains supplementary material, which is available to authorized users.

## Background

Dementia is a common disorder with major medical, economic and societal costs. The most frequent cause of dementia is Alzheimer’s disease (AD). Key neuropathological hallmarks of the AD brain are diffuse and neuritic extracellular amyloid plaques - often surrounded by dystrophic neurites - and intracellular neurofibrillary tangles. Although the aetiological mechanisms underlying these neuropathological changes remain unclear, the disease is thought to be multifactorial, resulting from complex interactions between genetic, lifestyle and environmental factors [1]. By 2050 it is estimated that the number of patients with AD worldwide will quadruple from the current number of 36 million [2].

Although AD is thought to be primarily a neurodegenerative disease, several experimental and clinical observations have suggested that vascular factors may play a role in disease pathogenesis and progression [3–5]. Prospective cohort studies have reported associations between vascular risk factors (VRF) and dementia including hypertension [6] and diabetes mellitus (DM) [7]. Case-control studies have linked a wide range of VRF with disease risk, including hypertension, DM, high cholesterol, atrial fibrillation, smoking, obesity and low physical activity.

Not only have VRF been associated with disease risk, but it has also been suggested that their presence accelerates disease progression [8]. This implies that control of VRF could have an impact on disease progression in the individual patient with AD, and may offer a useful secondary prevention treatment strategy [5]. Furthermore, it has been suggested that some drugs used to treat VRF, such as statins, may have specific pleotrophic protective effects in AD.

Before implementing the widespread treatment of VRF as part of routine clinical care in AD, it is important to show that their treatment really does reduce disease progression and impact on quality of life. To assess this we performed a systematic review to determine whether treatment of VRF is associated with improved clinical outcome in patients with already diagnosed AD. We included both randomised controlled trials (RCTs) and observational studies.

## Methods

Studies containing information on the treatment of VRF in patients with a diagnosis of AD were identified using a defined search strategy. RCTs and observational studies were included.

### Pre-specified search strategy

PubMed, MEDLINE, Embase, CENTRAL (Cochrane Library), DARE (Database of Abstracts of Reviews of Effects) and BIOSIS databases (conference abstract or proceedings) were searched between 1 January 1966 and 22 March 2014.

Search terms were (dementia OR "Alzheimer's Disease" OR Alzheimer OR "demented patients" OR "cognitive decline progression" OR "post-dementia cognitive change" OR "complications in dementia") AND (vascular risk factor OR predictors OR hypertension OR diabetes mellitus OR diabetes OR smoking OR hyperlipidaemia OR hypercholesterolaemia OR cholesterol OR "alcohol intake" OR overweight OR obesity) AND (treatment OR therapy OR control OR contribution OR antihypertensives OR hypoglycemic agents OR insulin treatment OR "smoking cessation" OR "alcohol intake reduction" OR "hypolipidemic agents" OR statins OR diet OR "weight loss" OR exercise OR "physical activity").

The search was limited to articles on humans, in English and Italian languages, and full papers. Abstracts were reviewed and articles potentially meeting inclusion criteria identified. References lists and reviews were hand searched. To exclude duplicate papers, Reference Manager 12 was used.

Articles were included if they fulfilled the following criteria: five patients or more; AD dementia (according to diagnostic criteria); VRFs (hypertension, DM, hyperlipidaemia, smoking, overweight and no exercise) defined according to reported criteria; AD progression with outcome measures (for example, Mini Mental Score Examination (MMSE) for cognition, Activities of Daily Living (ADL) and Modified Rankin Scale for disability); studies with a mixture of dementia types where it was possible to separate out the data on the AD patients; studies with a mixture of patients with AD and of mild cognitive impairment (MCI) where it was possible to separate the two groups.

Studies that dealt exclusively with laboratory or neuroimaging surrogate markers (for example, white matter hyperintensities on magnetic resonance imaging, cerebral blood flow measurements, cerebrospinal fluid markers or neuropathology) were not included.

### Data extraction and management

All studies meeting the inclusion criteria were independently assessed by two authors. In the event of disagreement or divergent analysis, a consensus was achieved by discussion between authors. If eligible data were available for a subset of study patients, the subset fitting the inclusion criteria was included. For duplicate data among studies, the article with the largest number of patients and/or longest follow-up was included.

Extracted data from articles meeting inclusion criteria were inserted into a standard proforma. The following information was collected: type of study (prospective, observational, RCT and retrospective studies); number of patients; recruitment of consecutive subjects; screening criteria for AD diagnosis; type of intervention or drug for each VRF; outcome measures instrument; follow-up or treatment duration; impact of vascular conditions on cognitive decline or progression of dementia; concomitant use of drugs for dementia; complications, institutionalisation and mortality.

Quality of data, including the presence of possible bias, was recorded and inserted into a standard proforma.

## Results

The search strategy retrieved 11,992 abstract articles from PubMed (5,170), MEDLINE (1,917), Embase (3,621), CENTRAL (1,217), DARE (50), and BIOSIS (17). Abstract screening identified 135 studies and, after reading full-text papers and identifying additional papers from reviews and reference lists, 25 of these studies met all inclusion criteria. Among these, 11 were RCTs and 14 were observational studies. Results are presented separately for these two study types. Observational studies were only included if they reported treatment of VRFs; studies in which the relationship between the presence of VRF, rather than their treatment, and AD progression were not included.

### Randomised controlled trials

Table 1 summarises the main features of the RCTs. Studies are hereafter reviewed according to risk factor treatment.

**Table 1:** **Randomised controlled trials that assessed the treatment of vascular risk factors in patients with a diagnosis of Alzheimer’s disease**

**Vascular risk factors**	**First author, reference**	**Population**	**Diagnosis criteria**	**Treatment arm (n, drug/daily)**	**Control arm (n)**	**Duration of follow-up (months)**	**Measurement instrument**
**Vascular care package**	**Richard** ***et al.*** **2009** [9]	123 AD (with co-existent cerebrovascular disease)	NINCDS-ADRDA	58 vascular care (aspirin, advice on smoking cessation, weight loss and exercise)	65 placebo	24	IDDD, MMSE, RMBPC, measures of poor outcome
**Hypertension**	**Ohrui** ***et al.*** **2004** [10 **]**	162 AD	NINCDS-ADRDA	51 brain-penetrating ACE-I	53 non-brain penetrating inhibitor versus 58 calcium-channel blocker	12	MMSE
	**Kume** ***et al.*** **2012** [11 **]**	20 AD	NINCDS-ADRDA, DSM IV	10 telmisartan (40 to 80 mg)	10 amlodipine (5 to 10 mg)	6	MMSE, ADAS-JCog, WMS-R
**Diabetes mellitus**	**Watson** ***et al.*** **2005** [20 **]**	21 mild AD	NINCDS-ADRDA	14 rosiglitazone (4 mg)	7 placebo	4, 6	Buschke Selective Reminding Test, Story Recall, SCWI, TMT, category fluency
	**Hanyu** ***et al.*** **2009** [21 **]**	26 AD	NINCDS-ADRDA	12 pioglitazone (15, 30 mg)	14 placebo	6	MMSE, IDDD, RMBPC
	**Sato** ***et al.*** **2011** [22 **]**	42 mild AD	NINCDS-ADRDA	21 pioglitazone (15 to 30 mg)	21 placebo	6	MMSE, ADAS-JCog, WMS-R, FAB
	**Risner** ***et al.*** **2006** [23 **]**	511 mild-to moderate probable AD	NINCDS-ADRDA	389 rosiglitazone (2, 4 or 8 mg)	122 placebo	6	ADAS-Cog, CIBIC+
	**Gold** ***et al.*** **2010** [24 **]**	581 AD	NINCDS-ADRDA	331 rosiglitazone (2 or 8 mg)	166 placebo	6	ADAS-Cog, CIBIC+
**Hypercholesterolaemia**	**Sparks** ***et al.*** **2005** [30 **]**	67 AD	NINCDS-ADRDA	32 atorvastatin (80 mg)	31 placebo	12	MMSE, ADAS-Cog, CGIC
	**Simons** ***et al.*** **2002** [31 **]**	44 probable AD	NINCDS-ADRDA	24 simvastatin (80 mg)	20 placebo	6.5	MMSE, ADAS-Cog
	**Feldman** ***et al.*** **2010** [33 **]**	640 AD	NINCDS-ADRDA	297 atorvastatin (80 mg)	317 placebo	18	MMSE, ADAS-Cog, ADCS-CGIC NPI, CDR-SB, ADFACS

ACE-I, angiotensin converting enzyme inhibitors; AD, Alzheimer’s disease; ADAS-Cog, Assessment Scale - Cognitive; ADAS-JCog, Assessment Scale - Cognitive Subscale (Japanese version); ADCS-CGIC, Alzheimer’s Disease Cooperative Study Clinical Global Impression of Change; ADFACS, Alzheimer’s Disease Functional Assessment and Change Scale; CDR-SB, Clinical Dementia Rating - Sum of Boxes; CIBIC+, Clinician’s Interview-Based Impression of Change Plus Caregiver Input; DSM-IV, Diagnostic and Statistical Manual of Mental Disorders, Fourth Edition; FAB, Frontal Assessment Battery; IDDD, Interview for Deterioration in Daily activities in Dementia; MMSE, Mini Mental Score Examination; RMBPC, Revised Memory and Behavioural Problems Checklist (Additional file 1); SCWI, Stroop Color-Word Interference; TMT, Trail-Making Test; WMS-R, Wechsler Memory Scale Revised.

The NINCDS-ADRDA Alzheimer's Criteria: National Institute of Neurological and Communicative Disorders and Stroke and the Alzheimer's Disease and Related Disorders Association.

#### Vascular care package addressing multiple risk factors

One RCT examined the effectiveness of a vascular care package that included aspirin, folic acid and pyridoxine drug therapy as well as advice on smoking cessation, weight loss and exercise [9]. Within this trial, 123 patients with mild AD with neuroimaging evidence of co-existent cerebrovascular disease (white matter hyperintensities or infarcts) were randomised and there was a two-year follow-up. Eleven patients died during follow-up and 18 dropped out. Significant differences in homocysteine and cholesterol occurred, but there was no difference, not even a trend, in the primary endpoint of disability or in secondary endpoints of MMSE or the Revised Memory and Behavioural Problems Checklist (Additional file 1) [9].

#### Hypertension treatment

We found no RCTs comparing antihypertensive treatment with placebo, although we identified two RCTs that compared the effectiveness of different antihypertensive agents in patients with AD.

One trial examined the hypothesis that brain-penetrating angiotensin converting enzyme inhibitors (ACE-I) would slow the rate of cognitive decline in patients with mild to moderate AD with hypertension [10]. This was on the rationale that certain components of the renin-angiotensin system (RAS) may have a role in learning and memory processes. In this trial, 162 patients were randomly assigned on an open basis to one of three treatment options: a brain-penetrating ACE-I (perindopril or captopril), a non-brain penetrating inhibitor (enalapril or imidapril), or a calcium antagonist (nifedipine or nilvadipine). Mean baseline MMSE values were 19.3, 20.7 and 20.5 in the three groups respectively. There was no difference in blood pressure between the three groups. The mean decline in the primary endpoint of the MMSE during the one-year follow-up was significantly lower in the groups treated with a brain-penetrating ACE-I (0.6, standard error (SE) = 0.1) than those in the other two groups: 4.6 (SE = 0.3) and 4.9 (SE = 0.3) respectively [10].

In a small study looking primarily at cerebral blood flow, 20 patients with AD were randomised to open therapy with telmisartan, an angiotensin receptor blocker (ARB), or amlodipine for six months. Cognition did not change in the telmisartan group but declined in the amlodipine group [11].

#### Diabetes mellitus treatment

The only RCTs of diabetic treatment are of peroxisome proliferator-activated receptor gamma (PPARγ) agonists, and have examined whether this particular class of drug has a protective effect rather than whether tighter diabetic control itself improves outcome. PPARγ agonists increase glucose sensitivity, regulate lipid metabolism and promote mitochondrial biogenesis [12,13]. They also exhibit robust anti-inflammatory actions via their ability to suppress NF-κB-dependent gene expression [14,15]. AD is typified by impaired glucose utilization in the brain and a glial-mediated inflammatory response, suggesting the potential utility of these agents in the treatment of AD [14–16]. Studies in murine models of AD demonstrated that rosiglitazone lowers amyloid plaque burden, reduces vascular and plaque-associated inflammation, attenuates loss of synaptic connectivity, and improves memory and cognition [14,17–19]. They have been tested both in patients with AD and diabetes, and patients with AD but without diabetes.

A pilot study suggested the PPARγ agonist rosiglitazone improved cognition in patients with mild-to-moderate AD [20]. Another small open study in 32 patients with both mild-to-moderate AD (or amnestic MCI) and diabetes mellitus not on insulin randomised patients between the PPARγ agonist pioglitazone or no additional treatment. A significant decrease in the AD Assessment Scale - Cognitive Subscale Japanese version (ADAS-JCog) and increase in the Wechsler Memory Scale Revised (WMS-R), but no change in MMSE, was found in the pioglitazone group [21]. Another small study in 42 patients with mild AD and type II DM primarily looked at regional cerebral blood flow but also included data on cognitive outcome, and suggested a protective effect of pioglitazone [22]. Patients were randomly assigned to open treatment with 15 or 30 mg daily pioglitazone in addition to their previous oral hypoglycaemic treatments. After six months, scores on the the MMSE, ADAS-JCog and WMS-R logical memory-I improved significantly in the pioglitazone group, while the ADAS-JCog worsened significantly in the control group. Neither group showed any significant change in the Frontal Assessment Battery and category fluency [22].

However, larger trials in patients with AD without diabetes have not replicated these positive results. A phase 2 trial in 511 non-diabetic patients with mild-to-moderate AD randomised participants between placebo or 2, 4 or 8 mg rosiglitazone [23]. There were no statistically significant differences in the primary endpoints (ADAS-Cog and Clinician’s Interview-Based Impression of Change Plus Caregiver Input (CIBIC+)) after 24 weeks’ treatment. Results were also stratified by ApoE genotype in a subgroup (n = 323); there was a significant interaction between ApoE epsilon4 allele status and ADAS-Cog (*P* = 0.014) with ApoE4-negative individuals appearing to show an improvement in response to rosiglitazone, whereas ApoE epsilon4 allele carriers showed no improvement and some decline was noted. But this interaction with ApoE status was not confirmed in a subsequent study. This double-blind phase 2 trial randomised 581 individuals with mild-to-moderate AD without diabetes to once-daily placebo, 2 mg or 8 mg rosiglitazone, placebo, or donepezil as a positive control [24]. At week 24, there was no significant difference from placebo in change from baseline in the ADAS-Cog score and CIBIC + with either rosiglitazone dose in the 50% of individuals who were ApoE epsilon4-negative, or overall [24].

#### Statin treatment

Statin (3-hydroxy-3-methylglutaryl-coenzyme A reductase inhibitors) treatment might act via a general cardiovascular protective effect due to cholesterol lowering or via a more specific effect on AD pathology. Cholesterol modulates the processing of amyloid precursor protein (APP)-related Aβ production *in vitro* and in animal model studies [25,26]. AD neuropathologic lesions are demonstrable in patients who have coronary artery disease and no dementia [27]. Reducing cholesterol through the use of statins appears to affect the processing of APP and the production of Aβ. A diet high in cholesterol increased Aβ accumulation and AD-related pathology in the transgenic mouse model, with relatively little change in brain cholesterol [28], and feeding a high-cholesterol diet to rabbits has also produced Aβ deposits in the hippocampus [29].

A single-centre study randomised 67 patients with mild-to-moderate AD (MMSE score of 12 TO 28) to either atorvastatin or placebo on a double-blind basis for one year [30]. A significant positive effect on ADAS-Cog performance occurred after six months of atorvastatin therapy compared with placebo, although the difference was not quite significant at one year [30]. Another small study of 44 patients with probable AD of mild-to-moderate severity (MMSE score 12 to 26), which was primarily looking at the effects of stains on cerebrospinal fluid markers, assessed cognition as a secondary outcome [31]. Patients were randomised to placebo or simvastatin and the simvastatin group had less decline in MMSE score during follow-up [31].

A *post hoc* analysis was conducted on data pooled from three double-blind, placebo-controlled, clinical trials of galantamine in patients with AD divided into four treatment groups: statin plus galantamine (n = 42), statin alone (n = 50), galantamine alone (n = 614), or neither galantamine nor statin (n = 619) [32]. Galantamine was associated with a significant beneficial effect on cognitive status, and although there was no significant benefit from statins (*P* = 0.083), there was a trend which led the authors to suggest further studies were needed [32].

By contrast, a subsequent larger international multi-centre, double-blind, randomised, parallel-group study failed to confirm these benefits [33]. The authors examined the use of atorvastatin in patients without an indication for statin, that is, no cardiovascular disease and low-density lipoprotein cholesterol levels on study entry between 95 and 195 mg/dL [33]. The 640 participants randomised had mild-to-moderate probable AD (MMSE 13 to 25), were aged 50 to 90 years, and were all taking 10 mg donepezil daily. They were randomised to 80 mg/day atorvastatin or placebo for 72 weeks. There were no significant differences in the co-primary endpoints of ADAS-Cog score or global function assessed by the Alzheimer’s Disease Cooperative Study Clinical Global Impression of Change. The proportion discontinuing treatment was 34.1% in the statin group and 24.5% in the placebo group, with those discontinuations felt to be related to therapy in 11.5% and 1.5% respectively. The authors suggest a limitation is that statin therapy may be more useful in those patients with AD patients and co-existing cardiovascular disease and/or high cholesterol level [33].

### Observational studies

A number of observational studies have evaluated whether the treatment of VRF is associated with a slower progression of cognitive decline or better outcome in AD patients (Table 2), although it is notable that for some VRF such as smoking and obesity we could find no data.

**Table 2:** Observational studies that evaluated whether the treatment of vascular risk factors is associated with a slower progression in patients with Alzheimer’s disease

**Vascular risk factors**	**Study**	**Population**	**Diagnosis criteria**	**Treatment arm and comparator (if any) (n, drug/daily)**	**Duration of follow-up (months)**	**Measurement instrument**
**Vascular care package**	Deschaintre *et al.* 2009 [34]	280 consecutive AD	NINCDS-ADRDA, DSM IV	208 treated (119 with some VRF treated and 89 with all VRF treated) versus 72 without treatment	27	MMSE
	Rosenberg *et al.* 2008 [35]	216 AD	NINCDS-ADRDA, DSM III R	Cardiovascular medication (ACE-I, β-blockers, calcium ion channel blockers, diuretics, statins, nitrates, platelet inhibitors or digoxin)	36	CDR-Sum
**Hypertension**	Razay *et al.* 2009 [36]	141 AD	NINCDS-ADRDA, NINDS-AIREN	Antihypertensive medications	60	CAMCOG
	Duron *et al.* 2009 [37]	321 consecutive AD	NINCDS-ADRDA, DSM IV	127 treated with antihypertensive drugs (calcium channel blockers, β-blockers, ACE-I, diuretics, ARB) versus 149 not treated	34.1	MMSE
	Bellew *et al.* 2004 [38]	719 mild-to- severe AD	NINCDS-ADRDA	Antihypertensive drugs	6	MMSE, ADAS-Cog
	Li *et al.* 2010 [39]	12574 AD	ICD 9	3,227 treated with lisinopril versus 476 with ARB versus 8,871 other cardiovascular drugs	48	Admission to nursing home
		12879 AD		3,333 treated with lisinopril versus 491 with ARB versus 9,055 other cardiovascular drugs		Mortality
	Hajjar *et al.* 2008 [40]	62 AD	NINCDS-ADRDA	15 treated with ACE-I versus 47 untreated	6	MMSE, CDT, DO, IADL, SCB
	Soto *et al.* 2013 [41]	616 mild-to-moderate AD	NINCDS-ADRDA	61 treated with ACE-I versus 189 with other antihypertensive drugs versus 309 without antihypertensive drugs	48	MMSE
	Ellul *et al.* 2007 [42]	224 probable AD	NINCDS-ADRDA	92 treated with antihypertensive drugs (including 20 with ACE-I and 2 with ARB), 12 with statins and 10 with anti-diabetic drugs	12	GDS
	Kehoe *et al.* 2013 [43]	3905 AD	Oxford Medical Information System or Read codes and prescriptions	1,323 treated with ACE-I versus 265 ARB versus 2,315 other antihypertensive drugs	120	Hospitalization and mortality
**Diabetes mellitus**	Plastino *et al.* 2010 [44]	104 AD	DSM IV	49 oral anti-diabetic drugs versus 55 insulin oral + anti-diabetic drugs	12	MMSE, CGI
**Hypercholesterolaemia**	Masse *et al.* 2005 [45]	234 AD	NINCDS-ADRDA	129 were dyslipaemic treated with LLAs (47% with statins) versus 105 untreated	34.8	MMSE
	Ellul *et al.* 2007 [42]	224 probable AD	NINCDS-ADRDA	92 treated with antihypertensive drugs (including 20 with ACE-I and 2 with ARB), 12 with statins and 10 with anti-diabetic drugs	12	GDS
	Padala *et al.* 2012 [48]	12 AD	-	Statins [atorvastatin (10, 20, 40, 80 mg), simvastatin (10, 40 mg), fluvastatin (20, 40 mg), pravastatin (20 mg), rosuvastatin (10 mg), lovastatin (40 mg)]	3 (1.5 discontinuation and 1.5 re-challenge)	MMSE, CERAD, ADL, IADL

ACE-I, angiotensin converting enzyme inhibitors; AD, Alzheimer’s disease; ADAS-Cog, AD Assessment Scale-Cognitive; ADL, Activities of Daily Living; ARB, angiotensin receptor blockers; β-blockers, β-blocking anti-adrenergics; CAMCOG, Cambridge Cognitive Examination; CDR-Sum, Clinical Dementia Rating Sum of Boxes; CDT, Clock Draw Test; CERAD, Consortium to Establish a Registry for Alzheimer’s Disease; CGI, Clinical Global Impression; DO, Digit Ordering; DSM III R, Diagnostic and Statistical Manual of Mental Disorders, Third Edition, Revision; GDS, Global Deterioration Scale; IADL, Instrumental Activities of Daily Living; MMSE, Mini Mental Score Examination; SCB, Screen for Caregiver Burden.

ICD- International Statistical Classification of Diseases and Related Health Problems. Usually just ICD would be used.

NINDS-AIREN National Institute of Neurological Disorders and Stroke and Association Internationale pour la Recherché et l'Enseignement en Neurosciences.

NINCDS-ADRDA. as previously.

#### Vascular care package addressing multiple risk factors

One study identified 280 patients from a memory clinic who had AD with no evidence of cerebrovascular disease and followed them up for six months [34]. Each VRF (high blood pressure, dyslipidaemia, DM, smoking) was considered treated if the patient received a specific medication. MMSE decline was slower for patients with all VRF treated compared with no VRF treated. There was a non-significant trend in patients with only some VRF treated. Although limited by the small numbers in each group, analysis of individual VRF found a significant effect only for the treatment of dyslipidaemia with either a statin or a fibrate [34].

In the Dementia Progression Study of the Cache County Study on Memory, Health and Aging, 216 individuals with incident AD were followed for a mean of three years [35]. The Clinical Dementia Rating Sum of Boxes (CDR-Sum) increased an average of 1.69 points annually, indicating a steady decline in functioning. After adjustment for demographic variables and the baseline presence of cardiovascular conditions, use of statins (*P* = 0.03) and beta-blockers (*P* = 0.04) were associated with a slower annual rate of increase in CDR-Sum of 0.75 and 0.68 points respectively, while diuretic use was associated with a faster rate of increase in CDR-Sum (*P* = 0.01; 0.96 points annually) [35].

#### Hypertension treatment

A number of studies have examined whether ant-hypertensive therapy is associated with reduced cognitive decline in AD. Some studies have included only hypertensive patients [36] whereas others also included normotensive individuals [37,38].

In the longitudinal OPTIMA study, among the 141 patients with AD in whom blood pressure was recorded, the rate of decline on Cambridge Cognitive Examination (CAMCOG) scores showed an inverted U-shape dependent on diastolic blood pressure. The use of any antihypertensive medication in those with AD was related to significantly better CAMCOG scores (*P* = 0.008) [36].

In a prospectively collected database of 321 patients with AD and hypertension with a mean follow-up of 34 months, cognitive function was assessed yearly by MMSE [37]. Fifty-four per cent of patients received at least one antihypertensive drug while 33% of those patients without antihypertensive treatment were hypertensive. Medication included different classes of drugs. MMSE was significantly higher among patients using antihypertensive drugs compared to those without antihypertensive treatment after adjustment for main confounders (19.0 versus 17.5, *P* <0.0001 at three years) [37].

A case-control study investigated the association between hypertension and cognitive decline in 719 patients diagnosed with AD who had been randomly assigned to the placebo arm of a clinical trial and followed-up for six months [38]. Eighty per cent had hypertension at baseline, defined as a past history, treatment or raised blood pressure. After controlling for baseline disease severity, patients with AD and hypertension were more likely to have increased cognitive decline with an odds ratio of 1.6. Secondary analysis suggested this effect was confined to younger patients (below 65 years). Treatment with antihypertensive medication appeared to have no effect on the rate of cognitive decline in those patients with AD and hypertension [38].

It has been suggested that drugs blocking the RAS, both ACE-I and ARB, might be particularly effective at preventing cognitive decline in AD; ARB may have had beneficial effects on cognition in some studies in patients without AD [39]. In a small study, 15 patients with both AD and hypertension treated with ACE-I were compared with 47 patients, of whom 43% were hypertensive, who were not treated [40]. Over a six-month follow-up, patients receiving ACE-I had a slower rate of decline in digit forward and Instrumental Activities of Daily Living scale, and an improved measure of caregiver burden after adjusting for other risks factors [40].

A further study evaluated 686 patients with AD, of whom 75% had hypertension [41]. Sixty-one were continuous users of ACE-I, 59 used ACE-I intermittently, 189 were users of other antihypertensive drugs, and 309 had never used antihypertensive drugs. The four-year decline in MMSE was 6.4, 7.9, 8.8 and 10.2 respectively. In a subgroup analysis, the 118 participants who had continuously or intermittently used ACE-I had a significantly lower decline compared with the 498 who had never used ACE-I (7.5 versus 9.7; *P* = 0.03) [41].

A study in 224 patients associated a wide variety of drugs with progression measured as a change in the Global Deterioration Scale. About half of the patients were on heart and antihypertensive drugs. As part of the analysis they found a protective effect of ACE-I [42].

A large study used the US Veterans database to examine the hypothesis that inhibition of the RAS might have a specific effect on dementia and that ARB treatment might be more effective than ACE-I [39]. The authors looked at progression of dementia in those with AD at baseline, with progression defined as death or admission to a nursing home. Patients on an ARB, on the ACE-I lisinopril, and on cardiovascular comparators (excluding an ARB, ACE-I or statin) were compared; the proportion with hypertension in each group was 93%, 91% and 80% respectively. Compared with the cardiovascular comparator, ARB in patients with pre-existing AD were associated with a significantly lower risk of admission to a nursing home (0.51; 95% confidence interval, 0.36 to 0.72) and death (0.83; 95% confidence interval, 0.71 to 0.97). ARB exhibited a dose-response as well as additive effects in combination with ACE-I [39].

In the large UK-based General Practice Research database it was hypothesised that the rates of progression to hospitalisation or death would be lower for patients with AD treated with an ARB compared to patients on other antihypertensive drugs through the reduction of angiotensin II signalling [43]. In 3,905 patients with AD, neither mortality or hospitalisation rates with ARB were different from those in patients treated with other antihypertensive drugs. Unexpectedly, ACE-I were associated with a significantly higher mortality, but not with any increase in hospitalisation [43].

#### Diabetes mellitus treatment

We could find no studies addressing the effect of diabetic therapy or control on outcome in AD, but one looked at possible protective effects of insulin therapy. Cognitive decline was compared between patients with mild-to-moderate AD and DM treated with insulin (n = 55) and those on oral hypoglycaemic agents alone (n = 49) [44]. At 12 months, mean MMSE decreased in those treated with oral hypoglycaemic agents (20.4 ± 4.1 versus 18.2 ± 3.6; *P* = 0.001), but remained stable in insulin-treated patients (21.9 ± 5.1 versus 21.2 ± 3.9; *P* = 1.03). No analysis of glucose control was performed and therefore it is unclear whether this difference related to better diabetic control or other effects of insulin [44].

#### Statin treatment

We could find no studies examining the effect of statins exclusively in patients with AD and hyperlipidaemia but found three studies that evaluated the effects of statin treatment in patients with AD in which the diagnosis of hypercholesterolaemia was not present in all patients. Interpretation is complex because some studies addressed both concerns as to whether short-term statin therapy may impair cognition, whereas others looked at protective effects over longer periods of follow-up.

A three-year observational study followed 342 patients with AD (MMSE 21.3 at entry) [45]. Patients were classified into those with dyslipaemia and treated with lipid-lowering agents (n = 129; 47% with statins), those who had untreated hyperlipidaemia (n = 105), and those who were normolipidaemic (n = 108) [45]. Patients treated with lipid-lowering agents had a slower decline on the MMSE (1.5 point/year, *P* = 0.01) than patients with untreated dyslipidaemia (2.4 points/year) and normolipidaemic patients (2.6 points/year) [45].

A study in 224 patients associated a wide variety of drugs with progression in AD. Only 12 (5%) were on statins, but this group had less decline on the Global Deterioration Scale [42].

The US Food and Drug Administration has added safety warnings to statins concerning confusion and memory loss [46]. Initial evidence of such adverse events came from case reports describing subjective and reversible worsening of cognition in individuals using statins, although none of those reports included objective cognitive measures [47]. Also case series are reported in which patients with MCI or dementia had a significant improvement in their MMSE score when statins were discontinued [48]. In 12 patients with AD, the short-term effects of statin withdrawal were studied in a 12-week prospective non-blinded study involving a six-week withdrawal phase and a six-week challenge phase [48]. A specific aim was to address concerns that statins might be associated with short-term memory impairment. There was an improvement in MMSE scores (+1.9 [3.0], *P* = 0.014) with discontinuation of statins and a decrease in MMSE scores (−1.9 [2.7], *P* = 0.007) after re-challenge [48]. Two prospective studies showed minor declines in cognition of uncertain significance in adults with hyperlipidaemia treated with statins [49,50].

## Discussion

How to treat VRF in this patient group is a common question facing clinicians, and if such treatment does have an effect on slowing disease progression it could have a major population impact due to the high prevalence of AD. A clear treatment benefit would mandate a systematic search for cardiovascular risk factors in this patient group. However, this is a patient group who may already be on other medications and in whom compliance can be difficult [51–54], and therefore it is important to avoid giving ineffective treatments. There are no clear guidelines as to optimal management in this area and therefore we carried out this systematic review.

Although there is substantial evidence that VRF are associated with an increased risk of AD, few studies have examined the effect of treatment of VRF, either as a package or individually, on progression of disease in patients with established AD.

The majority of studies we found were small. The interpretation is further complicated because a number of studies tested whether specific classes of drugs that alter AD pathology in animal models were effective, rather than testing whether treatment of the specific risk factor *per se* altered disease progression.

We found only 11 RCTs addressing this area and of these two compared different drug classes rather than determining whether treatment of the risk factor itself altered outcome. Sample sizes of thousands with follow-up of usually two to three years or more have been required to show effectiveness of risk factor treatment in secondary prevention of stroke; one might expect that similar sample sizes and follow-up would be required to determine whether VRF treatment alters progression in AD. Slightly more data was available from observational studies but again these suffered from relatively small sample sizes and many were retrospective analyses of pre-existing datasets.

One approach is to provide a vascular care package where all common VRF are treated. Two observational studies, each in approximately 200 patients with follow-up of six months and three years respectively, did suggest that such an approach might be associated with delayed progression [34,35]. However, such observational studies suffer from the potential bias that those patients being treated may be those who are felt to have a better prognosis and therefore have been given treatment for VRF. Only one small RCT examined this approach, and found no treatment effect [9]. Much larger sample sizes are required to definitely determine whether a package of VRF treatment will delay disease progression.

Considerable evidence suggests that hypertension is associated with an increased risk of AD [6], but there is much less data determining whether treatment of hypertension delays progression in patients with established disease. Observational data in a total of approximately 1,000 patients suggests that treatment may be associated with reduced progression. To date, no RCTs have examined this question.

There has been more interest in whether specific classes of antihypertensive drugs may have particular benefit. In particular, it has been hypothesised that drugs blocking the RAS, both ACE-I and ARB, might have specific benefits in AD. One large study of the US Veterans database [39] found ARB use appeared to be associated with improved outcome, and this study and some other smaller studies have suggested that ACE-I may also be beneficial. The relative benefit of RAS system blockers has received some support from small RCTs but further data is required before their routine use in AD can be recommended.

We found very little data on whether intensive control of diabetes is associated with reduced progression of AD. It has been suggested that the PPARγ agonists may have specific benefits in AD, perhaps by anti-inflammatory effects. This has led to these drugs, used as treatments in diabetes, to be tested in RCTs in AD. They have been given to patients with and without a diagnosis of diabetes. Although smaller studies suggested benefit, two larger trials in patients with no diabetes have not replicated these results [23,24].

Statin therapy could potentially reduce AD progression via its cholesterol-lowering effect and a reduction in vascular damage. It may have a more specific effect in AD, perhaps by altering processing of APP and production of Aβ. A relatively small number of observational studies provide some support for a benefit treatment effect, as do small RCTs. However, the only larger RCT performed in this area, which recruited 640 patients who were treated for 72 weeks, found no beneficial effect of atorvastatin [33].

Taken together, the available observational data raises the possibility that treating VRF could alter the rate of decline in AD. However, RCT data is not yet available to support this hypothesis and to alter clinical practice.

One might expect the magnitude of benefit would be no greater than that seen in secondary prevention of cardiovascular disease including stroke. If that is the case, there needs to be a paradigm shift in clinical trials addressing this issue. Much larger sample sizes are required, in the thousands or even tens of thousands as have been recruited to cardiovascular trials, and the duration of follow-up needs to be a number of years. Several cardiovascular trials, such as for statin therapy [55], have not shown separation between the treatment and placebo arms until after one year. Because the timescale to show benefit is likely to be a few years, it may be most practical to perform trials in patients with early AD. One potentially attractive option would be to perform trials using a VRF package including treatment of common risk factors such as hypertension, DM, hypercholesterolaemia and smoking cessation.

Studies need to address a number of other important potential confounding factors. Population-based studies have shown that many individuals have a mixed dementia with pathological features of both AD and vascular disease. One might expect treatment of VRF to have a more beneficial effect in this group, compared with ‘pure’ AD without vascular changes. Stratification by the presence or absence of neuroimaging changes such as leukoaraiosis and lacunar infarction on brain imaging may prove useful in investigating this area further.

## Conclusions

Considerable observational evidence has associated VRF with AD, raising the possibility that treating VRF could alter the rate of decline in AD. In this systematic review, we found that although some smaller studies suggest there may be a treatment effect, studies have been largely underpowered and do not provide sufficient data to change clinical practice.

What does a clinician do when faced with a patient with AD and VRF? One option is to treat all possible VRF on the assumption it may do good and is unlikely to do harm. Although superficially attractive, this approach is not ideal. Prescribing large numbers of ineffective drugs has significant disadvantages, in addition to the cost. A study found that patients with AD are routinely prescribed at least five drugs, and many are prescribed even more, in an attempt by providers to optimise disease state control [51]. In this predominantly elderly group with impaired cognition, compliance is often poor and the potential for misdosage high. Forty-one per cent of patients with AD on cardiovascular drugs were not taking their drugs regularly [52]. Furthermore, although widely used, drugs to treat VRF are not uncommonly associated with side effects, particularly in the elderly population most affected by AD, which can reduce quality of life.

Until the evidence base is stronger, a reasonable option is to treat VRF intensively if there is evidence of co-existent cardiovascular disease including both myocardial infarction and cerebrovascular disease. This could include evidence of cerebrovascular disease on brain imaging. But large, adequately powered trials are needed, ideally in the early stages of AD and in MCI, to determine whether treating VRF does delay progression in patients without overt cerebrovascular disease. If these are positive, this would have a major impact on the way AD is treated, and mean that protocols to screen patients with AD to detect and treat cardiovascular risk factors would need to be established.

## Additional file

Additional file 1:
**PRISMA 2009 Checklist.**

## Abbreviations

ACE-I: angiotensin-converting enzyme inhibitors
AD: Alzheimer’s disease
ADAS-Cog: Alzheimer’s Disease Assessment Scale-Cognitive
ADAS-JCog: Alzheimer’s Disease Assessment Scale-Cognitive Japanese version
ADL: Activities of Daily Living
APP: amyloid precursor protein
ARB: angiotensin receptor blockers
CAMCOG: Cambridge Cognitive Examination
CDR-Sum: Clinical Dementia Rating Sum of Boxes
CIBIC+: Clinician’s Interview-Based Impression of Change Plus Caregiver Input
DM: diabetes mellitus
MCI: mild cognitive impairment
MMSE: Mini Mental Score Examination
NK-κB: nuclear factor kappa-light-chain-enhancer of activated B cells
PPAR: peroxisome proliferator-activated receptor
RAS: renin-angiotensin system
RCT: randomised controlled trial
VRF: vascular risk factors
WMS-R: Wechsler Memory Scale Revised
